# Association of blood eicosapentaenoic acid levels with intracerebral hemorrhage during the COVID-19 pandemic: preliminary experience from a single-center in Japan

**DOI:** 10.1186/s12883-022-02657-7

**Published:** 2022-04-05

**Authors:** Kenichiro Hira, Yuji Ueno, Nobukazu Miyamoto, Sho Nakajima, Chikage Kijima, Nobutaka Hattori

**Affiliations:** Department of Neurology, Juntendo University School of Medicine, 2-1-1 Hongo, Bunkyo-ku, Tokyo, 113-8421 Japan

**Keywords:** COVID-19, Intracerebral hemorrhage, Eicosapentaenoic acid, Dietary habits

## Abstract

**Background:**

The COVID-19 pandemic has forced lockdowns and declarations of states of emergency, resulting in marked changes to daily life such as dietary habits in many countries. Though serum omega-3 polyunsaturated fatty acids levels have been shown to be useful markers for recurrent vascular events or worse prognosis in cardiovascular diseases and ischemic stroke, the relationship between serum omega-3 PUFA levels and the occurrence of intracerebral hemorrhage has essentially been unknown. We explored the association of serum omega-3 polyunsaturated fatty acids with intracerebral hemorrhage during the COVID-19 pandemic.

**Methods:**

Participants comprised patients admitted to Juntendo University Hospital (Tokyo, Japan) with intracerebral hemorrhage between January 1, 2016 and April 30, 2020. Clinical characteristics including serum omega-3 polyunsaturated fatty acid levels were compared between patients developing intracerebral hemorrhage during the period from January 1, 2016 to February 29, 2020, and the subsequent COVID-19 pandemic period (March 1 to April 30, 2020). Clinical characteristics independently related to intracerebral hemorrhage during the COVID-19 pandemic were analyzed by comparing these two cohorts of intracerebral hemorrhage patients in different periods.

**Results:**

A total of 103 patients (age, 67.0 ± 13.9 years; 67 males) with intracerebral hemorrhage were enrolled. Intracerebral hemorrhage developed in 91 patients before and 12 patients during the COVID-19 pandemic. Monthly averages of intracerebral hemorrhage patients admitted to our hospital during and before the COVID-19 pandemic were 6 and 1.82, respectively. Serum eicosapentaenoic acid levels were significantly lower in intracerebral hemorrhage patients during the COVID-19 pandemic than before (31.87 ± 12.93 μg/ml vs. 63.74 ± 43.29 μg/ml, *p* = 0.007). Multiple logistic regression analysis showed that, compared to before the COVID-19 pandemic, dyslipidemia (odds ratio 0.163, 95% confidence interval 0.031–0.852; *p* = 0.032) and eicosapentaenoic acid levels (odds ratio 0.947, 95% confidence interval 0.901–0.994; *p* = 0.029) were associated with intracerebral hemorrhage during the COVID-19 pandemic.

**Conclusions:**

From our preliminary results, low eicosapentaenoic acid levels were linked with intracerebral hemorrhage during the COVID-19 pandemic. Low levels of eicosapentaenoic acid might be an endogenous surrogate marker for intracerebral hemorrhage during the COVID-19 pandemic.

## Background

On March 11, 2020, the World Health Organization warned that coronavirus disease 2019 (COVID-19) had become a pandemic. The COVID-19 pandemic forced many countries to enforce lockdowns and declare states of emergency, resulting in marked changes to daily life, such as disrupting exercise, affecting dietary habits and alcohol consumption, and increasing mental distress and anxiety [1, 2]. Although many studies have helped in elucidating the pathological mechanisms of COVID-19, the associations of such social impacts of COVID-19 with the development of illnesses have not been fully elucidated.

Intracerebral hemorrhage (ICH) is the second most common subtype of stroke, accounting for 10–20% of all strokes, and is more common in Asian countries than in Western countries [3]. ICH involves the rupture of small cerebral arteries, causing critical clinical symptoms usually leading to severe disability or death. Age, male sex, and atherosclerotic vascular risk factors (particularly hypertension) have been identified as risk factors for ICH [4]. High alcohol consumption, poor dietary habits, and emotional distress have also been linked to ICH, and lifestyle-related and psychological factors could thus be important contributors to the development of ICH [4–6]. Although ischemic stroke was comorbid with COVID-19 in 0.9–2.4% of cases [7, 8], ICH associated with COVID-19 has been much less described, with only isolated case reports and a small number of case series published to date [9–12].

In Japan, a special measures law to combat the COVID-19 pandemic was approved by the government on March 13, 2020, and a state of emergency was declared on April 7 in Tokyo and six prefectures, subsequently spreading to the entirety of Japan. Before declaring the state of emergency, the number of COVID-19 patients had been increasing in Tokyo in March 2020 (https://stopcovid19.metro.tokyo.lg.jp/en); self-regulation of going out and teleworking were recommended, and lifestyle and psychological alterations were suggested to have occurred among residents of Tokyo. A previous work indicated that the lockdown forced by the COVID-19 pandemic altered dietary habits [1, 2]. Especially for individuals with overweight and obesity, the lockdown affected healthy dietary choices [1]. Meanwhile, large-scale cohort studies showed that the Japanese were fish-eating people and had higher omega-3 polyunsaturated fatty acid (PUFA) levels in the blood than people in other countries [13–15]. So far, many studies have shown the association of serum omega-3 levels with the development of cardiovascular diseases and ischemic stroke [16–19], but the relationship between serum omega-3 PUFA levels and occurrence of ICH was essentially unknown. We focused on the association of serum omega-3 PUFA levels such as eicosapentaenoic acid (EPA) and docosahexaenoic acid (DHA) with ICH, especially during the COVID-19 pandemic. Furthermore, the current preliminary study compared the profile of omega-3 PUFAs of ICH patients before and during the COVID-19 pandemic, and factors related to ICH during the COVID-19 pandemic were analyzed.

## Materials and methods

### Study participants

This case series was based on an analysis of data acquired from the prospective stroke registry of consecutive patients admitted to the Department of Neurology at Juntendo University Hospital (Bunkyo-ku, Tokyo, Japan) during the study period from January 1, 2016 to April 30, 2020. All patients with ICH underwent blood collection, electrocardiography, and computed tomography (CT) and magnetic resonance imaging (MRI) of the brain. Stroke severity was assessed using National Institutes of Health Stroke Scale (NIHSS) scores on admission, at the time of exacerbation, and at discharge. Disability in activities of daily living before admission and at discharge was assessed using the modified Rankin Scale.

Our institution is located in central Tokyo, and in consideration of the daily numbers of new-onset patients with COVID-19 (https://stopcovid19.metro.tokyo.lg.jp/en), the dates of approval for the special measures law to address the COVID-19 pandemic (March 13, 2020) and the declaration of the state of emergency in Tokyo (April 7, 2020), we defined the COVID-19 pandemic period as March 1 and April 30, 2020, which was corresponded to the first COVID-19 pandemic in Japan. We then compared clinical characteristics between patients who developed ICH during the period from January 1, 2016 to February 29, 2020 and the subsequent period of the COVID-19 pandemic (March 1 to April 30, 2020). Comparing ICH patients before and during the COVID-19 pandemic, clinical characteristics independently related to ICH during the COVID-19 pandemic were analyzed. During the COVID-19 pandemic, ICH patients with COVID-19 infection were excluded according to general examinations by physicians [20], chest CT, and positive polymerase chain reaction (PCR) results for SARS-CoV-2. The present study was approved by the ethics committee of Juntendo University Hospital. As clinical information obtained from medical records was used for all analyses in this study, the need to obtain written informed consent from each patient was waived. This study was conducted in accordance with the Declaration of Helsinki.

### Risk factors

We defined atherosclerotic vascular risk factors as follows: 1) hypertension [systolic blood pressure > 140 mmHg or diastolic blood pressure > 90 mmHg (in subacute phase; ≥14 days from admission), history of hypertension or use of antihypertensive agents]; 2) diabetes mellitus (use of oral hypoglycemic agents or insulin, or hemoglobin A1c ≥6.5%); 3) dyslipidemia [use of antihyperlipidemic agents, serum low-density lipoprotein cholesterol (LDL-C) ≥140 mg/dl, high-density lipoprotein cholesterol (HDL-C) < 40 mg/dl, or triglycerides ≥150 mg/dl]; 4) chronic kidney disease (CKD) [estimated glomerular filtration rate (eGFR) < 60 ml/min/1.73 m^2^ as calculated using the following equation for Japanese adults approved by the Japanese Society of Nephrology: eGFR = 194*serum creatinine^-1.094^*age^-0.287^*0.739 (if female)]; 5) smoking (current); 6) ischemic heart disease (defined as a history of angina pectoris or myocardial infarction); or 7) atrial fibrillation (history of atrial fibrillation or electrocardiographic finding).

### Radiological investigations

CT and MRI of the brain were performed on admission with the patient placed in a supine position. CT of the brain and chest was performed using an Aquilion PRIME CT scanner. Scan parameters were as follows: head – 120 kV; 300 mAs (automatic exposure control, AEC); collimation, 5 mm; pitch factor, 0.825; matrix, 512 × 512; and chest – 120 kV; 25–175 mAs (AEC); collimation, 5 mm; pitch factor, 0.825; matrix, 512 × 512. No contrast agents were administered. All images were transmitted to the postprocessing workstation for reconstruction by high-resolution algorithms and conventional algorithms. We diagnosed cerebral hemorrhage as focal hyperdense lesions > 10 mm in diameter on brain CT. To estimate the volume of cerebral hemorrhage, maximum length (in centimeters) was multiplied by the width perpendicular to maximum length on the same head CT slice, and by the number of slices multiplied by slice thickness, then divided by two [21].

MRI was performed using a 3-T MR scanner equipped for single-shot echo planar imaging (MAGNETOM Prisma; Siemens Healthineers, Munich, Germany) and including diffusion-weighted imaging (DWI). Total imaging time was approximately 15 min. A standard DWI sequence (repetition time (TR), 4000 ms; echo time (TE), 60 ms; field of view, 230 × 230 mm; matrix, 186 × 186; section thickness, 4 mm; intersection gap, 0 mm), fluid-attenuated inversion recovery (FLAIR) sequence (TR, 9000 ms; TE, 114 ms; field of view, 230 × 230 mm; matrix, 320 × 320; section thickness, 5 mm; intersection gap, 1 mm; two signals acquired), and MRA sequence (TR, 21 ms; TE, 3.69 ms; field of view, 180 × 180 mm; matrix, 320 × 320; section thickness, 0.55 mm; intersection gap, 5.5 mm) were obtained. Cerebral microbleeds were identified using gradient-echo T2*-weighted MRI sequences (TR, 410 ms; TE, 12 ms; field of view, 230 × 230 mm; matrix, 320 × 320; section thickness, 5 mm; intersection gap, 1 mm; two signals acquired). According to previous studies, periventricular hyperintensity and deep and subcortical white matter hyperintensity were defined as high-intensity lesions on axial FLAIR images [22].

### Biochemical blood testing

Serum EPA and DHA were assayed by gas chromatography at an external laboratory (SRL Inc., Tokyo, Japan). Other laboratory data including leukocyte count, LDL-C, HDL-C, triglycerides, eGFR, blood sugar, hemoglobin A1c, high-sensitivity C-reactive protein, prothrombin time-international normalized ratio (PT-INR), activated partial thromboplastin time, and D-dimer were analyzed. Blood examinations were conducted within 24 h of admission or referral to our department.

### Statistical analysis

Numerical values are reported as mean ± standard deviation. Baseline characteristics, laboratory data, clinical symptoms, and radiological observations related to ICH were compared between patients who developed ICH before and during the COVID-19 pandemic. Data were analyzed using the chi-squared test for categorical variables and the Mann-Whitney test for nonparametric analyses. To elucidate the factors related to ICH during the COVID-19 pandemic, all variables from ICH patients with values of *p* < 0.1 on univariate analyses were entered into multiple logistic regression analysis and compared to values of ICH patients before the COVID-19 pandemic. Each serum omega-3 PUFA level, including EPA, DHA, EPA + DHA, and ratios of EPA/arachidonic acid (AA), DHA/AA, and EPA + DHA/AA, showing a value of *p* < 0.1 was entered into different multiple logistic regression models separately, because these covariates confound each other. Values of < 0.05 were considered significant. All data analyses were conducted using SPSS for Mac version 26.0 (SPSS Inc., Chicago, IL, USA).

### Data availability

Data in support of the present findings are available from the corresponding author on reasonable request.

## Results

### Study population

A total of 103 consecutive patients with cerebral hemorrhage were admitted during the study period. Mean age was 67.0 ± 13.9 years, and 67 males (65%) were enrolled. Mean baseline NIHSS score was 8.5 ± 9.1. Numbers of ICH patients during and before the COVID-19 pandemic were 12 and 91, respectively. Monthly average numbers of ICH patients admitted to our hospital during and before the COVID-19 pandemic were 6 and 1.82, respectively. None of the 12 patients with ICH during the COVID-19 pandemic showed any evidence of COVID-19 infection according to general examinations, chest CT, and PCR testing for SARS-CoV-2.

### Baseline characteristics of patients with ICH occurring before and during the COVID-19 pandemic

Table 1 lists baseline characteristics for each group of patients with ICH admitted before the COVID-19 pandemic, and patients with ICH admitted to our department during the COVID-19 pandemic. Patients with ICH during the COVID-19 pandemic were younger (57.8 ± 14.3 years) than those before the COVID-19 pandemic (68.3 ± 13.5 years; *p* = 0.027). Diastolic blood pressure on admission was relatively higher in patients with ICH during the COVID-19 pandemic (104.8 ± 24.6 mmHg) than in those with ICH before the pandemic (93.7 ± 22.1 mmHg; *p* = 0.057). Dyslipidemia was relatively less common in patients with ICH during the COVID-19 pandemic (33% vs. 66%; *p* = 0.061). No significant differences in atherosclerotic vascular risk factors or use of anti-thrombotic drugs before ICH were seen between groups. No significant differences in clinical symptoms including NIHSS on admission, neurological deterioration, or in-hospital death were found. There was no significant difference between ICH before and during the COVID-19 pandemic in latency from ICH onset to radiological imaging (290 ± 334 min vs. 149 ± 113 min, *p* = 0.106). On CT and MRI, size, location, and expansion of hematoma, intraventricular penetration, and white matter diseases did not differ between groups (Table 1).

**Table 1:** Baseline characteristics, clinical symptoms and laboratory and MRI findings of patients with ICH before and during the COVID-19 pandemic

	Total	COVID-19 pandemic		
Variables	***N*** = 103	Previous ***n*** = 91, 88%	Midst ***n*** = 12, 12%	***P***
Mean age, y	67.0 ± 13.9	68.3 ± 13.5	57.8 ± 14.3	0.027
Male gender, n (%)	67 (65)	58 (64)	9 (75)	0.655
BMI, kg/m^2^, a	23.0 ± 4.1	23.0 ± 4.0	23.0 ± 5.2	0.85
Systolic BP, mmHg	172.3 ± 52.7	171.5 ± 54.7	178.3 ± 34.6	0.441
Diastolic BP, mmHg	95.0 ± 22.4	93.7 ± 22.1	104.8 ± 24.6	0.057
**Risk factors,** n (%)				
Hypertension	93 (90)	84 (92)	9 (75)	0.166
Diabetes mellitus	20 (19)	19 (21)	1 (8)	0.519
Dyslipidemia	64 (62)	60 (66)	4 (33)	0.061
Smoking	24 (23)	20 (22)	4 (33)	0.609
Chronic kidney disease	20 (19)	19 (21)	1 (8)	0.519
Atrial fibrillation	11 (11)	10 (11)	1 (8)	0.828
Ischemic heart disease	11 (11)	10 (11)	1 (8)	0.828
History of stroke	17 (17)	16 (18)	1 (8)	0.691
**Preceding treatment,** n (%)				
Oral anticoagulants	11 (11)	10 (11)	1 (8)	0.828
Antiplatelet agents	19 (18)	18 (20)	1 (8)	0.572
**Clinical symptoms**				
mRS before ICH	0.5 ± 1.1	0.6 ± 1.1	0.2 ± 0.6	0.189
NIHSS on admission	8.5 ± 9.1	8.4 ± 9.4	9.4 ± 6.4	0.285
mRS at discharge	2.5 ± 1.9	2.4 ± 1.9	2.7 ± 1.8	0.646
NIHSS at discharge	6.6 ± 11.6	6.6 ± 11.8	6.6 ± 10.3	0.386
Neurological deterioration with NIHSS ≥4	7 (7)	6 (7)	1 (8)	0.7
Death during hospitalization	6 (6)	6 (7)	0 (0)	0.794
**Latency from ICH onset to radiological imaging**, b	269 ± 314	290 ± 334	149 ± 113	0.106
**CT findings**				
Hematoma volume (cm^3^)	16.8 ± 26.5	17.1 ± 27.9	14.6 ± 11.1	0.371
Hematoma expansion	7 (7)	10 (11)	2 (17)	0.922
Intraventricular penetration	34 (33)	31 (34)	3 (25)	0.763
**MRI findings**				
Location of hemorrhage				0.245
Thalamus	28 (27)	26 (29)	2 (17)	
Putamen	38 (37)	30 (33)	8 (67)	
Subcortical	26 (25)	25 (27)	1 (8)	
Infratentorium	7 (7)	6 (7)	1 (8)	
Others	4 (4)	4 (4)	0 (0)	
PVH	1.3 ± 0.9	1.3 ± 0.9	1.3 ± 0.9	0.69
DSWMH	1.1 ± 0.9	1.1 ± 0.9	0.8 ± 0.6	0.208
CMBs	67 (68)	59 (69)	8 (67)	0.844
**Latency from ICH onset to blood sampling**, b	244 ± 310	266 ± 330	123 ± 100	0.239
**Laboratory findings**				
LDL-C, mg/dl, a	111.1 ± 41.6	111.8 ± 42.9	106.3 ± 28.9	0.783
HDL-C, mg/dl, a	56.2 ± 19.5	56.2 ± 20.0	56.6 ± 14.7	0.835
Triglyceride, mg/dl, a	127.0 ± 99.6	123.9 ± 95.7	150.3 ± 126.8	0.763
eGFR (ml/min)	78.0 ± 31.1	76.8 ± 31.3	86.8 ± 29.4	0.149
BS (mg/dl)	129.9 ± 39.3	131.9 ± 41.3	114.4 ± 14.6	0.113
Hemoglobin A1c (%), c	5.8 ± 0.7	5.8 ± 0.7	5.7 ± 0.3	0.717
Hs-CRP (mg/dl)	0.4 ± 1.2	0.4 ± 1.3	0.2 ± 0.3	0.491
PT-INR, a	1.12 ± 0.40	1.13 ± 0.40	1.09 ± 0.43	0.025
APTT/Control, a	1.02 ± 0.19	1.02 ± 0.19	1.07 ± 0.19	0.253
D-dimer (μg/ml), a	4.2 ± 15.7	4.5 ± 16.6	1.4 ± 1.0	0.44

Chi-square test and the Mann−Whitney U test were used for comparison. *ICH* Intracerebral hemorrhage, *COVID-19* Coronavirus disease 2019, *BMI* Body mass index, *BP* Blood pressure, *LDL-C* Low-density lipoprotein cholesterol, *HDL-C* High-density lipoprotein cholesterol, *eGFR* Estimated glomerular filtration rate, *BS* Body sugar, *hs-CRP* High sensitive C reactive protein, *PT-INR* Prothrombin time-international normalized ratio, *APTT* Activated partial thromboplastin time, *NA* Not available, Chronic kidney disease was defined as eGFR < 60 ml/min/1.73 m^2^. Missing values: a, *n* = 1; b, *n* = 18; c, *n* = 2

### Laboratory data including serum levels of omega-3 PUFAs in patients with ICH before and during the COVID-19 pandemic

**Fig. 1:**
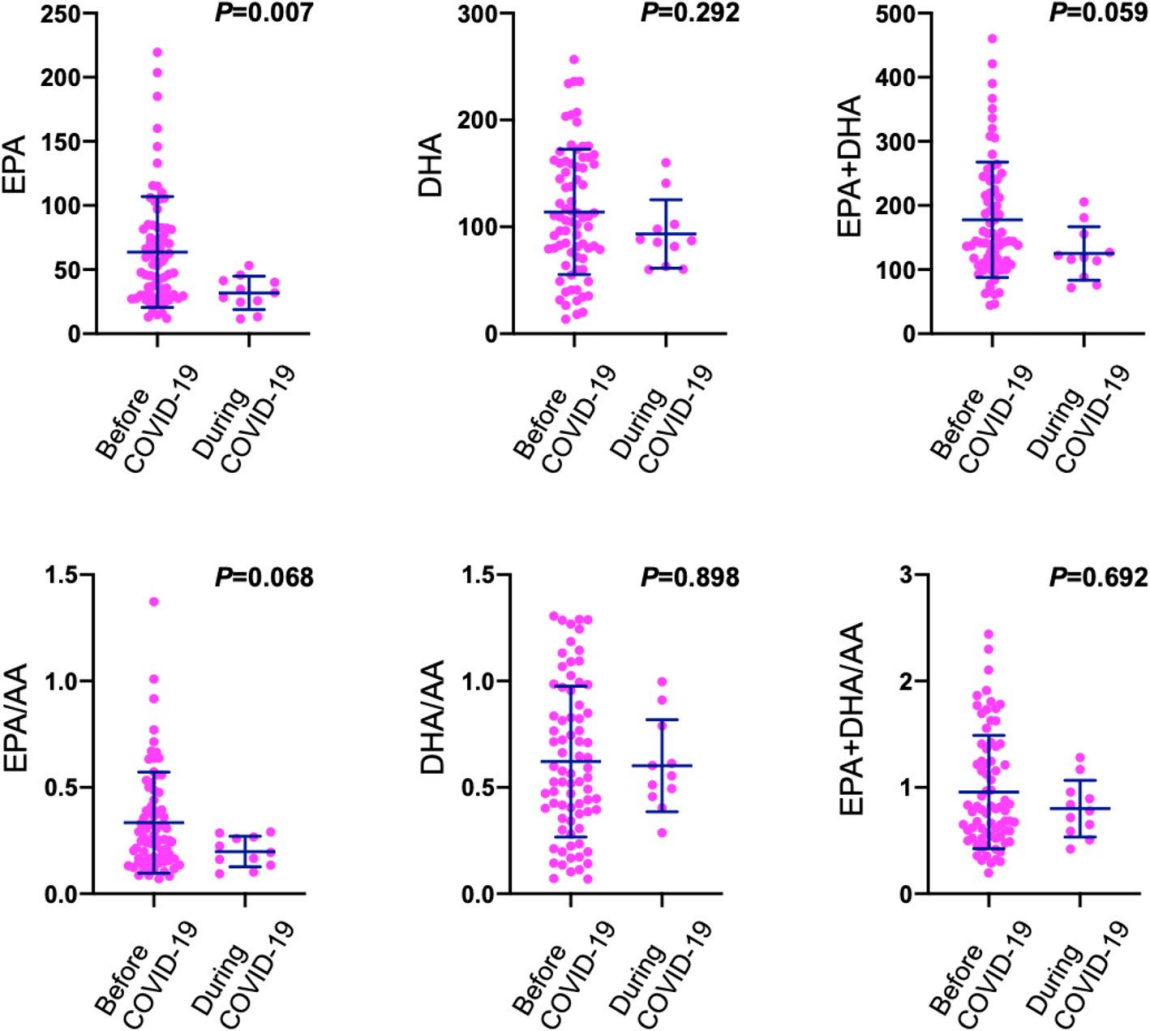
Comparison of serum omega-3 polyunsaturated fatty acid levels between ICH patients before and during the COVID-19 pandemic. Serum levels of EPA (63.74 ± 43.29 μg/ml vs. 31.87 ± 12.93 μg/ml), DHA (113.99 ± 58.58 μg/ml vs. 93.38 ± 31.91 μg/ml), EPA + DHA (175.42 ± 91.8 μg/ml vs. 125.25 ± 41.69 μg/ml), EPA/AA ratio (0.33 ± 0.24 vs. 0.20 ± 0.07), DHA/AA ratio (0.62 ± 0.35 vs. 0.60 ± 0.22), and EPA + DHA/AA ratio (0.96 ± 0.53 vs. 0.80 ± 0.27 in ICH patients before and during the COVID-19 pandemic, respectively (*n* = 87). The Mann-Whitney U test was used for comparison. ICH = intracerebral hemorrhage; EPA = eicosapentaenoic acid; DHA = docosahexaenoic acid; AA = arachidonic acid

There was no significant difference between ICH before and during the COVID-19 pandemic in latency from ICH onset to blood sampling (266 ± 330 min vs. 123 ± 100 min, *p* = 0.239). PT-INR was lower in ICH patients during the COVID-19 pandemic (1.09 ± 0.43 vs. 1.13 ± 0.40, *p* = 0.025). EPA levels were significantly lower in ICH patients during the COVID-19 pandemic than those in ICH patients before the COVID-19 pandemic (31.87 ± 12.93 μg/ml vs. 63.74 ± 43.29 μg/ml; *p* = 0.007) (Fig. 1). The sum of EPA and DHA and the EPA/AA ratio were relatively lower in ICH patients during the COVID-19 pandemic (EPA + DHA: 125.25 ± 41.69 μg/ml vs. 175.43 ± 91.8 μg/ml, *p* = 0.059; EPA/AA ratio: 0.20 ± 0.07 vs. 0.33 ± 0.24, *p* = 0.068) (Fig. 1).

### Underlying characteristics related to ICH during the COVID-19 pandemic

Age, dyslipidemia, diastolic blood pressure, and PT-INR were entered into multiple logistic regression analysis to determine independent factors related to the clinical characteristics of ICH developing during the COVID-19 pandemic. Of the omega-3 PUFAs, EPA, EPA + DHA, and EPA/DHA ratio had shown *p* < 0.1 on univariate analyses were separately entered into each model. Accordingly, EPA, EPA + DHA, and EPA/DHA ratio were entered into Models A, B, and C, respectively, while age, dyslipidemia, diastolic blood pressure, and PT-INR were entered equally into all Models. In Model A, dyslipidemia (odds ratio [OR] 0.163, 95% confidence interval [CI] 0.031–0.852; *p* = 0.032) and blood EPA levels (OR 0.947; 95%CI 0.901–0.994; *p* = 0.029) were independently associated with ICH during the COVID-19 pandemic (Table 2). In Models B and C, dyslipidemia (B – OR 0.206, 95%CI 0.052–0.806; *p* = 0.023; C – OR 0.185, 95%CI 0.039–0.884; *p* = 0.034, respectively) was significantly associated with ICH during the COVID-19 pandemic, while EPA + DHA (OR 0.995, 95%CI 0.987–1.003; *p* = 0.252) and EPA/AA ratio (OR 0.003, 95%CI 0.000–1.870; *p* = 0.077) were not.

**Table 2:** Logistic regression analysis for predictive factors associated with ICH during COVID-19 pandemic: Model A

	OR	95% CI	***P*** value
Age (per 1 y)	0.947	0.893–1.005	0.072
Dyslipidemia	0.163	0.031–0.852	0.032
Diastolic BP	1.006	0.975–1.037	0.718
PT-INR	4.531	0.354–58.038	0.245
EPA	0.947	0.901–0.994	0.029

All variables with a *P* < 0.1 on univariate analysis were entered into the logistic regression analysis. *ICH* Intracerebral hemorrhage, *COVID-19* Coronavirus disease 2019, *BP* Blood pressure, *PT-INR* Prothrombin time-international normalized ratio, *EPA* Eicosapentaenoic acid, *OR* Odds ratio, *CI* Confidence interval

## Discussion

The present study explored the profiles of omega-3 PUFAs among ICH before and during the COVID-19 pandemic and explored the underlying characteristics related to ICH during the COVID-19 pandemic in a single-center investigation. The salient finding of our preliminary study was that lower EPA levels were related to ICH during the COVID-19 pandemic compared to ICH before the COVID-19 pandemic.

SARS-CoV-2 could directly cause ICH in relation to endothelial injuries and disruption of the blood-brain barrier via ACE2 receptors, but the prevalence of ICH was much lower compared to ischemic stroke [23–25]. In our study, participants were negative for infection by SARS-CoV-2, and alternative pathogeneses other than direct effects of COVID-19 could therefore be involved in the development of ICH. In studies in Europe, alterations in dietary habits were not uncommon during the lockdown period, and the opportunity to eat healthy foods became difficult, which was more prominent in overweight and obese patients [1, 2]. Our data highlighted that serum EPA levels were independently related to ICH during the COVID-19 pandemic. Blood EPA levels represent the dietary intake of fish or omega-3 PUFAs, as shown in large-scale population studies [13–15]. Moreover, alcohol consumption, a traditional risk factor for ICH [4], was increased together with psychological burdens such as fear, stress, and anxiety during the COVID-19 pandemic [26, 27]. Social stress, anxiety, and depression are potential risk factors for hemorrhagic stroke [28, 29]. In our study, various social and lifestyle factors could be causes of ICH, but we did not assess psychological burden or dietary habits in ICH patients, and precise mechanisms of developing ICH in the COVID-19 pandemic are yet to be elucidated.

Meanwhile, the COVID-19 pandemic resulted in confusion in emergency medical facilities, such as refusal to accept ambulances due to insufficient preparation of medical systems for acute stroke patients in each facility and an unbalanced transport situation in Tokyo, particularly before the publication of Japanese Stroke Society-Protected Code Stroke (JSS-PCS, https://www.jsts.gr.jp/) on April 24, 2020 based on the PCS by Khosravani et al. [30]. Thus, ICH patients who were not accepted in other hospitals and were referred to our hospital might have increased. Conversely, a substantial reduction in acute stroke alerts by approximately 30% was reported in emergency departments in northwest Ohio in the United States during the COVID-19 pandemic, which might indicate a true reduction in stroke incidence or patients not seeking medical attention for emergencies during the COVID-19 pandemic [31]. Indeed, numbers of ICH patients admitted to our hospital during and before the COVID-19 pandemic were 12 in 2 months and 91 in 50 months, respectively, and thus the number of ICH patients admitted to our hospital during the COVID-19 pandemic increased compared to before. However, regional data in Tokyo to explore the incidence of ICH before and during COVID-19 pandemic are required.

The current study revealed the notable finding that lower levels of EPA were significantly associated with ICH during the COVID-19 pandemic. EPA competitively suppresses the formation of prostaglandin E2 from arachidonic acid, instead producing the less-inflammatory prostaglandin E3, and promotes inflammatory convergence via the production of E-series resolvins, as metabolites of EPA. EPA thereby suppresses platelet aggregation, inhibition of monocyte/neutrophil migration, and improvement of endothelial injury [32]. Although fish consumption might be positively related to hemorrhagic stroke, as shown among Greenland Inuit in 1980 [33], a recent meta-analysis did not show any risk of ICH after EPA consumption [34]. Furthermore, large-scale clinical trials using a high content of EPA did not show ICH as an adverse event, but reduced cardiovascular diseases and stroke [35, 36]. Of the serum levels of omega-3 PUFAs such as EPA, DHA and the ratios of EPA/AA and DHA/AA have been related to the risk of cardiovascular diseases [16–18]. In ischemic stroke, lower ratios of EPA/AA and DHA/AA were associated with the development of ischemic stroke in younger patients (< 65 years) compared to controls (< 65 years) and older stroke patients (≥65 years) [19]. On the other hand, it was shown that the ratio of EPA/AA, but not DHA/AA, was associated with cardiovascular events and stroke [18]. So far, the difference between EPA and DHA as a surrogate marker for cardiovascular diseases is yet to be elucidated. Meanwhile, a previous study enrolling Japanese subjects showed that serum EPA levels decreased with age [37]. Although ICH patients during the COVID-19 pandemic were younger than ICH patients before the COVID-19 pandemic, multiple logistic regression analysis showed a significant association of serum EPA levels, but not age, with ICH during the COVID-19 pandemic. Collectively, our preliminary data suggest that low EPA levels may reflect an endogenous surrogate marker for ICH during the COVID-19 pandemic, but one can not exclude the possibility of age-related effect for serum EPA levels.

Our data also indicated that dyslipidemia was negatively related to ICH during the COVID-19 pandemic. As for dyslipidemia as a risk factor for ICH, there is evidence that LDL-C < 80 mg/dl represents a risk for ICH in population-based studies [38]. However, ICH patients enrolled in the present study showed approximately 110 mg/dl. Due to the small sample size of ICH, further study is warranted.

Various limitations to this study should be considered. First, the number of ICH patients during the COVID-19 pandemic was small, and the period of the COVID-19 pandemic was short, without considering seasonal variations of ICH occurrence. Second, data from the present study were derived from a single center. Third, detailed data on dietary and alcohol habits in enrolled patients before the development of ICH were unavailable. Fourth, some laboratory data were missing. In particular, serum PUFA levels were analyzed in 87 patients. Accordingly, our data were based on preliminary experience from a single center, and a multicenter investigation or nationwide surveillance is warranted in the future.

## Conclusions

It was identified that low EPA levels were related to ICH during the COVID-19 pandemic in preliminary experience from a single center in Japan. Although we did not address the direct associations of serum EPA levels with age-related effect and dietary habits during the COVID-19 pandemic, EPA might be an endogenous surrogate marker for ICH during the COVID-19 pandemic.

## Data Availability

The datasets used and analyzed during the current study are available from the corresponding author upon reasonable request.

## Abbreviations

COVID-19: Coronavirus disease 2019
ICH: Intracerebral hemorrhage
PUFA: Polyunsaturated fatty acid
EPA: Eicosapentaenoic acid
DHA: Docosahexaenoic acid
CT: Computed tomography
MRI: Magnetic resonance imaging
NIHSS: National Institutes of Health Stroke Scale
PCR: Polymerase chain reaction
LDL-C: Low-density lipoprotein cholesterol
HDL-C: High-density lipoprotein cholesterol
CKD: Chronic kidney disease
eGFR: Estimated glomerular filtration rate
AEC: Automatic exposure control
DWI: Diffusion-weighted imaging
TR: Repetition time
TE: Echo time
FLAIR: Fluid-attenuated inversion recovery
PT-INR: Prothrombin time-international normalized ratio
AA: Arachidonic acid
OR: Odds ratio
CI: Confidence interval
